# How much can Mexican healthcare providers learn about breastfeeding through a semi-virtual training? A propensity score matching analysis

**DOI:** 10.1186/s13006-020-00297-6

**Published:** 2020-06-29

**Authors:** Mireya Vilar-Compte, Rafael Pérez-Escamilla, Meztli Moncada, Diana Flores

**Affiliations:** 1 Research Institute for Equitable Development EQUIDE, Universidad Iberoamericana, Mexico City, Mexico; 2 Department of Social and Behavioral Sciences, Yale School of Public Health, New Haven, CT USA

**Keywords:** Breastfeeding, Training, EsIAN, Mexico

## Abstract

**Background:**

Mexico has shown a worrisome decrease in breastfeeding indicators, especially in the lowest socioeconomic level. Improving breastfeeding protection, promotion, and support services through workforce development is a key area of intervention. The objective of this study is to assess the influence on breastfeeding knowledge and abilities of a semi-virtual training for primary healthcare providers assisting beneficiaries of PROSPERA in Mexico, which is one of the largest conditional cash-transfer programs in the world.

**Methods:**

Two independent cross-sectional samples of healthcare providers were drawn at baseline and post-intervention in three states of Mexico. Baseline data were collected among primary physicians, registered nurses and nurse technicians (i.e. unit of analysis) on July 2016 (*n* = 529) and post-training between March and April 2017 (*n* = 211). A 19-item telephone questionnaire assessed providers’ general knowledge about breastfeeding, breastfeeding benefits and clinical aspects of breastfeeding, clinical ability to solve problems and abilities to overcome breastfeeding challenges. The effects of the training were assessed through a propensity score matching (PSM) stratified by types of providers (i.e. physicians, registered nurses, nurse technicians).

**Results:**

The PSM analysis showed significant improvements among all providers in the general knowledge about breastfeeding (around 20 percentage points [pp]) and knowledge about breastfeeding benefits (approximately 50 pp). In addition, physicians improved their knowledge about clinical aspects of breastfeeding (7 pp), while registered nurses improved in their ability to solve breastfeeding problems (14 pp) and in helping mothers overcome breastfeeding challenges (12 pp).

**Conclusions:**

Promoting a breastfeeding enabling environment in Mexico to improve breastfeeding rates will require improving the knowledge and skills of healthcare providers. While a semi-virtual training showed large improvements in knowledge, developing skills among providers may require a more intensive approach.

## Background

Mexico is a middle-high income country that has experienced a worrisome decrease in breastfeeding indicators, especially among those in the lowest socioeconomic level and indigenous people. According to the Mexican National Health and Nutrition Survey (ENSANUT for its acronym in Spanish), the prevalence of exclusive breastfeeding (EBF) among those under 6 months of age was 14.4% in 2012, 8 percentage points lower than that reported in 2006 (22.3%) [1]. A recent analysis of breastfeeding protection, promotion, and support systems in Mexico underscored the need for in-service training to improve breastfeeding knowledge and abilities among healthcare providers [2]. This is consistent with recommendations to improve the coverage and quality of breastfeeding support services globally through workforce development to attain the breastfeeding goals set by the World Health Assembly, including at least 50% prevalence of exclusive breastfeeding by 2030 [3, 4].

During the former administration (2012–2018), the Mexican federal government implemented a nutrition training strategy targeting healthcare providers who worked with beneficiaries of the PROSPERA program, which was one of the largest conditional cash-transfer programs in the world. Formerly named *Progresa,* PROSPERA was initially launched in 1997 as a cash-transfer program conditional on children’s attendance to school, nutrition workshops and preventive medical services and checkups. PROSPERA aimed to strengthen the social rights and developmental capacities of low-income families to break the intergenerational poverty cycle, by simultaneously addressing three key elements for human capital formation: education, health, and nutrition [5]. In 2017, close to 7.2 million families were affiliated with the program, and their beneficiaries included 1,255,869 children under 5 years of age [6]. Given the size of this subpopulation and the importance of early childhood development for future health-related and cognitive outcomes [7], in 2014, the health and social development governmental agencies launched a maternal-child nutrition strategy called EsIAN (the initiative’s acronym in Spanish). EsIAN was designed to improve the nutritional and health-related outcomes of pregnant women and children under 5 years of age enrolled in PROSPERA.

EsIAN’s goals were to promote (i) breastfeeding and complementary feeding according to the World Health Organization (WHO) recommendations, (ii) the correct use of micronutrient fortified food supplementation, (iii) appropriate nutrition for pregnant and breastfeeding women, and (iv) the timely identification of gestational diabetes and hypertension during pregnancy. To achieve these objectives, three major interventions were implemented. First, all healthcare units serving PROSPERA beneficiaries were adequately equipped to perform nutritional assessments. Second, nutritional supplements and the criteria for prescribing them were reviewed. Finally, a training program was launched to improve the knowledge, abilities, and practices of healthcare providers in terms of nutrition, the use of micronutrient fortified food supplementation, and breastfeeding. The training followed a cascade model in which the National Institute of Health (INSP for its acronym in Spanish) trained health educators from the states (*n* = 32), who then trained primary physicians, registered nurses (RNs), and nurse technicians based at public primary healthcare clinics. The training was based on a semi-virtual approach involving initial face-to-face contact followed by electronic-based modules, and was designed on prior formative research [8].

The aims of this study were (i) to provide a detailed description of the baseline breastfeeding knowledge and abilities among primary physicians, RNs and nurse technicians working in clinics that served PROSPERA’s population, and (ii) to assess the influence of a semi-virtual training on providers’ breastfeeding knowledge and abilities. This innovative study has the potential to be highly impactful because it focuses on a promising approach to improve breastfeeding knowledge and abilities among healthcare providers serving highly socio-economically vulnerable populations enrolled in conditional cash-transfer programs.

## Methods

An evaluation was launched in 2016–17 to assess the influence(s) of the training intervention on nutrition, supplementation, and breastfeeding knowledge and abilities among healthcare providers in three states of Mexico (i.e. Chihuahua, Oaxaca, and Veracruz). As shown in Table 1, the selected states were diverse in terms of location, size, and social development [9–11]. The evaluation inquired about all the targeted topics of the training, however, the current analysis only discusses those related to breastfeeding.

**Table 1:** Characteristics of the intervention states in terms of location, size and development indicators

	State		
Characteristic	***Chihuahua***	***Oaxaca***	***Veracruz***
Location/Region	North	Southwest	Southeast
Size of the population (millions)	3.5	3.9	8.0
Poverty (% of the population)	30.6	70.0	62.2
Size of population enrolled in PROSPERA	480 thousand	1.6 million	2.5 million

Sources: CONEVAL, 2017; INEGI, 2015; PROSPERA, 2016

### Intervention

The implementation of the intervention in the evaluated states started in September 2016 when the first training cascade transitioned into the second training cascade. The frist training cascade involved training of state-level health officials in charge of training and education programs, while the second training cascade involved training of frontline providers. The intervention targeted primary care physicians, RNs and nurse technicians working in primary care clinics serving beneficiaries of the PROSPERA program. The training was based on the central premise that to achieve health promotion and disease prevention in the area of maternal-child health among mothers and children enrolled in PROPSERA, healthcare providers needed to improve their technical knowledge and communication abilities. Improvements in such knowledge and abilities were expected to increase their abilities to listen and understand beneficiaries’ issues and preferences, and consequently, to provide more effective advice to mothers.

The feasibility of the semi-virtual training was tested through formative research that confirmed providers had the technical skills and access to the electronic devices to complete the training. The content of the course was delivered through an electronic format – which could be online or through a USB or CD – and included visual elements, evidence-based guidelines, and case studies providing specific solutions to common lactation problems [12]. This format allowed to standardize the evidence-based information delivered to all healthcare providers, with the training cascade delivering the electronic course to frontline healthcare providers who received support from their trainers as they were completing the electronic course. This course format also allowed providers to complete the training at their own pace.

### Sample

Based on a before-and-after design, two independent cross-sectional samples of healthcare providers were drawn at baseline and post-intervention. Healthcare providers (i.e. primary physicians, RNs, nurse technicians) were the unit of analysis. Baseline data were collected in July 2016 and post-training data were collected between March and April 2017. This timeline allowed for providing enough time to detect improvements in breastfeeding knowledge and abilities if the training was indeed effective. The National Commission of Social Protection in Health provided a database with the names and telephone numbers of all PROSPERA health professionals in these states. Based on this list, at baseline and post-training, study participants were identified through a direct random sampling method (based on a computer algorithm), that was stratified by state, type of provider, and healthcare institution (e.g. state health clinics or IMSS-PROSPERA). Any healthcare provider working full-time in primary care clinics serving PROSPERA’s beneficiaries was eligible for inclusion.

### Data collection

To provide a detailed description of the knowledge and abilities about breastfeeding, a telephone survey was conducted with primary physicians and nurses in clinics serving PROSPERA’s beneficiaries at baseline and post-training. A telephone survey was a feasible strategy, as the telephone numbers were available for the roster of targeted healthcare providers. The survey was administered by trained interviewers who were blinded about the status of the training through a call center and lasted approximately 15 min. At baseline, 529 healthcare providers answered the questionnaire and 211 at post-intervention. The difference in sample size between the baseline and post-intervention groups was explained by the fact that during baseline, a consultant was hired to verify and track incorrect phone numbers, but this was not feasible at the post-data-collection phase because of resource constraints. Verbal consent was explicitly requested before the survey was conducted. To ensure data collection quality, a subsample of interviews were recorded and analyzed to assess errors in respondent’s eligibility, biases in procedures of data collection, and missing data and data patterns by the interviewer. The analyses suggested that data had adequate quality (reports are available upon request from corresponding author).

### Measures

The survey measured breastfeeding and human lactation knowledge and abilities through close-ended questions that only had one correct answer. The questionnaire included 19 items that were highly consistent with the breastfeeding training curriculum offered by EsIAN and was piloted in 2015. Some items inquired directly about specific evidence-based practices or knowledge, while other items were adapted from a pedagogical methodology based on vignettes presenting hypothetical cases to the healthcare providers [13, 14]. For example, a vignette was based on the following case *“Beatriz takes her 4 months old baby to a medical appointment. She is breastfeeding but she considers that she is not producing enough milk; hence, she is considering starting the baby on formula. Which of the following recommendations would you give to Beatriz? (i) As she is reporting a [breast milk] production issue, start the baby on formula right away (i.e. without probing further); (ii) [assume that] it is likely that the mother is currently undernourished, hence she should stop breastfeeding; or (iii) check the breastfeeding technique and correct any possible issue, and encourage the mother to keep breastfeeding”*. Vignettes have been used to assess service delivery quality, and have been valuable for quality improvement because they not only measure knowledge but also specific applied knowledge abilities [15].

The questionnaire assessed the knowledge and applied knowledge abilities of primary physicians and nurses in five areas: (i) general knowledge about breastfeeding based on WHO recommendations [3], definition of exclusive breastfeeding, perceptions about human milk substitutes, human milk extraction and storage, and introduction of complementary feeding; (ii) knowledge about maternal-infant health benefits related to breastfeeding; (iii) knowledge about clinical aspects of breastfeeding, including managing anemia among breastfeeding mothers, breastfeeding issues among malnourished mothers, and breastfeeding advice for diverse medical conditions and when taking medications; (iv) clinical abilities to help mothers who had problems breastfeeding exclusively, including self-report of insufficient milk production and maternal employment; and (v) ability to overcome breastfeeding challenges.

To assess the influence of the semi-virtual EsIAN training among healthcare providers, scores for each item, as well as for the five areas of breastfeeding knowledge and applied knowledge abilities were generated for baseline and post-training. The scores represented the percentage of correct answers. As the instruments used in the study were designed to measure the specifics of the training and had not been used in prior studies, they were piloted in a sample of 100 providers before data collection. The Cronbach’s alpha for the breastfeeding questionnaire was 0.75, which indicates adequate internal consistency.

To control for covariates, we examined healthcare provider characteristics including age, gender, professional category or level, and type of institution where the respondent was delivering care. Age was retained as a continuous variable, and gender was transformed into a dummy variable. Professional level was operationalized based on academic title and divided into three mutually exclusive categories: (i) primary physicians, e.g., medical doctors or recent medical school graduates completing their year of social service; (ii) registered nurses (RNs), e.g., nurses with university degrees; and (iii) nurse technicians, e.g., nurses without university degrees but with nurse technician certificates who normally helped RNs and physicians in clinics. The types of institutions employing these healthcare providers were classified either as public health insurance state services, which were decentralized services, or as IMSS-PROSPERA, which were centralized services that mostly serve rural areas. All institutions were public and served PROSPERA’s beneficiaries.

### Analysis

Descriptive statistics were estimated to compare the descriptive characteristics of the study samples at baseline and post-intervention. Bivariate analyses were performed for the 19-items that measured breastfeeding knowledge and abilities. Additionally, to assess the influence of the training, a propensity score matching analysis (PSM) was performed, a technique that allows matching pre- and post-intervention participants with similar characteristics. This statistical approach involved two steps. First, through a logit model, we estimated the probability of being in baseline or post-intervention as a function of a vector of covariates (i.e. age, gender, institution, stratum and state) by type of provider (i.e. primary physicians, registered nurses, nurse technicians). This step generated a propensity score *p(X*_*i*_*)* that allowed the identification of the “treated” and “controls”. In the second step, the average effect of the training (ATT) was computed, comparing scores at baseline and post-intervention by matching individuals with similar characteristics. The propensity score was calculated using Kernel matching, assuming independent observations with fixed weights and a set seed. Finally, to correct for any potential bias, we bootstrapped the ATT and computed the standard errors and 95% confidence intervals. The bootstrapping was estimated using 500 replications with the same data. Analysis were performed in STATA v.15 [16]; all *p*-values and confidence intervals were two-tailed.

The study was reviewed and approved by the Research Ethics Committee of Universidad Iberoamericana in Mexico City.

## Results

Table 2 shows the characteristics of the study sample at baseline and post-intervention. On average, the providers were 39 years old at baseline and 38 post-intervention. Close to one third were males in both periods. About one third of the providers were employed in rural clinics in both periods; slightly higher for post-intervention, but non-significant. There were some significant differences in the types of providers interviewed in each of the periods. At baseline around 48% of the providers were primary physicians, 25% RNs and 27% nurse technicians. At post-intervention there were fewer physicians (40%) and RNs (23%), and more nurse technicians (37%).

**Table 2:** Characteristics of the cross-sectional study samples at baseline and post-intervention

	***Baseline***		***Post-intervention***		
	Mean	SD	Mean	SD	*p*-value ^*a*^
Age	39.23	10.82	38.49	11.02	0.407
	**%**	**N**	**%**	**N**	***p*-value**^**b**^
**Sex**					
Male	28.73	152	29.86	63	0.761
Female	71.27	377	70.14	148	
**Type of Provider**					
Primary Physicians	47.83	253	40.28	85	0.031
Registered Nurses	24.95	132	22.75	48	
Nurse Technicians	27.22	144	36.97	78	
**Institution**					
IMSS-PROSPERA	26.65	141	40.76	86	0.000
State health clinics	73.35	388	59.24	125	
**State**					
Chihuahua	16.64	88	22.75	48	0.058
Oaxaca	34.03	180	36.49	77	
Veracruz	49.34	261	40.76	86	
**Stratum**					
Urban	68.24	361	62.09	131	0.109
Rural	31.76	168	37.91	80	
**Total**	**100.00**	**529**	**100.00**	**211**	

Note: *SD* standard deviation; ^a^ Mean difference t-test. ^b^ Pearson chi-square test

A bivariate analysis stratified by type of providers is summarized in Table 3. The aggregate score for general knowledge about breastfeeding was relatively low at baseline for all types of providers (between 61 and 67 out of 100) and post-training significantly improved to a similar extent across providers (i.e. between 18 and 24 percentage points [pp]). The score related to the knowledge of breastfeeding benefits was very low at baseline (between 22 and 26 out of 100) and improved substantially post training; 58 pp. more for physicians, 53 pp. more for RNs and 47 pp. more for nurse technicians. The score on knowledge about clinical aspects of breastfeeding was also very low at baseline (between 49 and 58 out of 100), and improved significantly only among physicians (7 pp) and nurse technicians (7 pp). On the other hand, the scores relating to abilities, only improved among physicians and RNs; primary physicians improved 6 pp. their clinical ability to solve breastfeeding problems, while RN’s improved their clinical ability to solve breastfeeding problems and their ability to help mothers to overcome breastfeeding challenges by 15 pp. and 14 pp., respectively.

**Table 3:** Scores on breastfeeding knowledge and abilities by type of healthcare provider. Baseline vs. post-intervention

		*Primary physicians*				*Registered nurses*				*Nurse technicians*			
		*Baseline*	*Post*	*Difference*	*p-value*	*Baseline*	*Post*	*Difference*	*p-value*	*Baseline*	*Post*	*Difference*	*p-value*
**I**	**General knowledge about breastfeeding**	67.32	88.43	21.10	0.00	61.62	85.76	24.15	0.00	63.43	81.84	18.41	0.00
1	Time of breastfeeding based on WHO recommendations	41.50	55.30	13.80	0.02	34.09	39.68	5.49	0.49	36.11	47.43	11.32	0.10
2	Introduction of complementary feeding	93.68	92.95	−0.73	0.81	91.67	93.75	2.08	0.64	92.36	88.46	−3.90	0.33
3	Definition of exclusive breastfeeding	87.75	92.94	5.19	0.13	82.58	97.92	15.34	0.00	84.03	87.18	3.15	0.53
4	Perception about human milk substitutes	91.30	97.65	6.34	0.00	79.55	95.83	16.29	0.00	84.03	87.18	3.15	0.53
5	Enough milk production to breastfeed their children	3.16	98.82	95.66	0.00	3.79	95.83	92.05	0.00	9.72	89.74	80.02	0.00
6	Human milk extraction and storage	86.56	92.94	6.38	0.07	78.03	91.67	13.64	0.01	74.30	91.02	16.72	0.00
**II**	**Knowledge about breastfeeding benefits**	22.43	80.59	58.15	0.00	25.57	78.13	52.56	0.00	25.35	72.12	46.77	0.00
7	Breastfeeding is related to less weight loss during the first months postpartum	44.66	50.59	5.92	0.34	45.45	33.33	−12.12	0.14	37.50	39.74	2.24	0.74
8	Breastfeeding is associated with lower risk of diabetes, breast and ovaries cancer	8.70	87.06	78.36	0.00	5.30	93.76	88.45	0.00	10.42	78.20	67.78	0.00
9	Breastfeeding may increase the risk of diarrheal infections in the infant	20.15	89.41	69.25	0.00	33.33	87.5	54.17	0.00	29.86	79.48	49.62	0.00
10	Breast milk does not provide all the nutrients and liquids that a baby needs during the first 6 months of life, so other foods and liquids must be given	16.20	95.29	79.09	0.00	18.18	97.92	79.73	0.00	23.61	91.02	67.41	0.00
**III**	**Knowledge about clinical aspects of breastfeeding**	57.80	64.41	6.60	0.01	56.63	54.69	−1.94	0.63	49.48	56.09	6.61	0.03
11	Managing anemia	97.23	92.94	−4.29	0.15	93.94	77.08	−16.86	0.01	90.27	85.89	4.38	0.32
12	Medicines during lactation	44.27	62.35	18.08	0.00	35.61	41.62	6.06	0.45	29.86	44.87	15.01	0.03
13	Breastfeeding advice for diverse medical conditions	63.64	58.82	−4.81	0.42	59.09	68.75	9.66	0.24	55.55	56.41	0.85	0.90
14	Malnourished mothers	26.09	43.53	17.44	0.00	37.88	31.25	−6.63	0.41	22.22	37.18	14.96	0.02
**IV**	**Clinical ability to solve problems**	85.11	90.98	5.86	0.02	79.55	94.44	14.90	0.00	79.17	83.76	4.59	0.18
15	Insufficient breast milk	79.05	90.58	11.53	0.00	67.42	95.83	28.41	0.00	70.83	88.46	17.62	0.00
16	Breastfeed and work	92.89	95.29	2.40	0.39	93.18	95.83	2.65	0.51	90.27	87.17	−3.09	0.50
17	Feed the baby with other foods instead of breast milk before 3 months of age	83.40	87.05	3.66	0.42	78.03	91.67	13.64	0.01	76.38	75.64	−0.74	0.90
**V**	**Ability to overcome breastfeeding challenges**	88.54	85.88	−2.66	0.42	80.68	94.79	14.11	0.00	78.81	71.79	−7.02	0.10
18	Risk of introducing other foods before 6 months of age	94.86	85.88	−8.98	0.02	93.18	93.75	0.57	0.89	92.36	73.07	−19.28	0.00
19	Most common cause for a child not getting enough breast milk	82.21	85.88	3.67	0.43	68.18	95.83	27.65	0.00	65.28	70.51	5.23	0.43

Due to the fact that baseline and post-intervention were independent random samples, PSM technique was used to control for potential unobserved differences between samples. Table 4 shows the standardized mean differences (SMD) between treated and untreated providers after the matching. To account for potential selection bias on missing observations, we corrected our treatment variable using doubly robust (DR) estimators. We did not find any significant difference on the standardized means between groups; i.e. SMD < 0.10 for all the characteristic after the matching. In addition, we observed the matching included all the observations except for two Treated-Nurse Technicians who were dropped due to not finding a common support. Thus, these results confirm the reliability of the matching.

**Table 4:** Balanced characteristics for Treated and Untreated groups after Kernel matching by type of provider

	***Primary Physicians***			***Registered Nurses***			***Nurse Technicians***		
	T	UT	SMD	T	UT	SMD	T	UT	SMD
Age	39.38	39.38	0.00	37.94	37.72	0.02	39.47	39.61	−0.01
**State**									
Chihuahua	0.15	0.15	0.01	0.22	0.22	0.00	0.21	0.21	−0.01
Oaxaca	0.38	0.36	0.05	0.30	0.29	0.02	0.39	0.38	0.02
Veracruz	0.46	0.49	−0.05	0.48	0.49	−0.03	0.41	0.41	−0.01
**Institution**									
IMSS-PROSPERA	0.69	0.70	−0.03	0.87	0.87	−0.02	0.54	0.53	0.02
State health clinics	0.31	0.30	0.03	0.13	0.13	0.02	0.46	0.47	−0.02
**Sex**									
Male	0.51	0.50	0.01	0.09	0.12	−0.07	0.11	0.11	0.00
Female	0.49	0.50	−0.01	0.91	0.88	0.07	0.89	0.89	0.00
**Stratum**									
Rural	0.32	0.34	−0.04	0.23	0.26	−0.05	0.38	0.39	−0.01
Urban	0.68	0.66	0.04	0.77	0.74	0.05	0.62	0.61	0.01
N	48	132		85	253		76	144	

Note: *T* Standardized Mean/Share of Treated; *UT* Standardized Mean/Share of Untreated; *SMD* Standardized Means Difference

Table 5 shows the results from the propensity score matching analysis. The score on breastfeeding general knowledge remained significant for all types of providers, and suggested that after the training, physicians improved their scores by 21 pp., RNs by 24 pp. and nurse technicians by 20 pp. The score improvements related to knowledge about breastfeeding benefits was also significant for all types of providers 59 pp. for physicians, 53 pp. for RNs, and 49 pp. among nurse technicians. Propensity score matching analysis showed that the clinical aspects of breastfeeding knowledge ATT score – which encompasses issues such as managing anemia among breastfeeding mothers, breastfeeding among malnourished mothers, and breastfeeding advice for diverse medical conditions and when taking medications – were only significant among physicians. On average physicians improved in 7 pp. their knowledge in clinical aspects of breastfeeding. By contrast, only RNs showed significant improvements in the two scores related to lactation management abilities. In the score related to clinical abilities to solve breastfeeding problems, RNs improved by 14 pp., and in the score linked to abilities to help mother overcome breastfeeding challenges they improved by 12 pp.

**Table 5:** Gains in breastfeeding knowledge and abilities between baseline and post-intervention by type of provider

	ATT ^**a**^	SE ^**b**^	CI [95%] ^**b**^	
**Primary Physicians**				
General knowledge about breastfeeding	20.85***	1.78	17.21	24.08
Knowledge about breastfeeding benefits	59.20***	2.68	54.23	64.39
Knowledge clinical aspects of breastfeeding	7.23**	2.93	1.49	13.08
Clinical ability to solve problems	4.36	2.66	−2.02	8.77
Ability to overcome breastfeeding challenges	−2.83	3.58	−11.76	3.16
**Registered nurses**				
General knowledge about breastfeeding	23.81***	3.07	17.60	29.37
Knowledge about breastfeeding benefits	52.65***	3.27	46.12	58.07
Knowledge clinical aspects of breastfeeding	−2.08	4.79	−10.40	8.14
Clinical ability to solve problems	14.39***	3.70	8.00	23.46
Ability to overcome breastfeeding challenges	12.07***	3.83	5.02	19.68
**Nurse technicians**				
General knowledge about breastfeeding	20.18***	2.59	15.16	26.18
Knowledge about breastfeeding benefits	49.27***	3.73	44.75	57.66
Knowledge clinical aspects of breastfeeding	6.47	3.68	−0.66	13.02
Clinical ability to solve problems	5.58	3.82	−1.49	12.58
Ability to overcome breastfeeding challenges	−7.00	5.20	−16.19	4.17

Notes: *ATT* Average Treatment effect for the Treated; *SE* Standard Error; *CI* 95% Confidence Interval; ^a^ Kernel matching for time of intervention; ^b^ Bootstrap coefficient estimated with 500 replications; Significance: ** *p*-value< 0.05, *** *p*-value < 0.01

## Discussion

This analysis is based on a unique study addressing breastfeeding knowledge and abilities among public primary healthcare providers from three contrasting states in Mexico. The findings documented that, at baseline, providers lacked basic knowledge and abilities to provide adequate breastfeeding counseling to mothers living under poverty conditions. This highlights the importance of a prior analysis highlighting the relevance of improving the coverage and quality of in-service breastfeeding training in Mexico [14].

The findings also suggest that a relatively easy-to-implement semi-virtual training intervention significantly improved breastfeeding knowledge among physicians, RNs, and nurse technicians working in primary clinics serving vulnerable families. This is a relevant finding, considering that prior studies have highlighted that improvements in breastfeeding knowledge scores among healthcare providers are significantly correlated with positive breastfeeding attitudes [17–19]. However, the analysis suggested that skill-related improvements were only achieved among RNs. This suggests that there is a need for a more intensive and hands-on training approach to further develop the abilities for breastfeeding problem solving among physicians and nurse technicians. Findings also raise the issue of whether different categories of providers should have differential roles in helping and counselling breastfeeding women.

Investment in increasing the knowledge and abilities of primary physicians and RNs seems highly relevant, as these are often the first health professionals that women have contact with after giving birth. Furthermore, this first contact is a key opportunity to promote and support breastfeeding [20, 21]. Hence, the training of healthcare provider is a relevant step toward improving breastfeeding outcomes. Prior research has highlighted that women who stop breastfeeding are often given incorrect advice by healthcare providers with limited evidence-based training and information, lack of clinical evidence-based reasoning, misconceptions, or incorrect assumptions [22–24]. It has also been reported that a healthcare provider’s lack of understanding toward extended breastfeeding can harm the patient-provider relationship [17, 25] resulting in early supplementation or abandonment of breastfeeding.

An important strength of our study was the use of a questionnaire with adequate internal consistency to assess the basic competency of healthcare providers who care for breastfeeding mothers and infants. One advantage of our study is its easy-to-apply and efficient telephone survey, which measured breastfeeding knowledge and applied knowledge abilities with acceptable internal consistency, allowing us to assess a large number of providers in a relatively short time. Specifically, our study documented that in a middle-income country, a telephone survey questionnaire is feasible for monitoring breastfeeding knowledge among healthcare providers, if a comprehensive sampling framework of providers (and their telephone numbers) is available.

Electronic resources have been used in different ways (i.e. online, as part of face-to-face courses) to enhance knowledge, attitudes and abilities about breastfeeding among healthcare professionals [26, 27]. Our study findings are consistent with those reported in a study in Milan, Italy in which an online course on lactation and infant feeding practices improved professional’s attitudes towards breastfeeding but led to minor changes on practices. There is an urgent need to systematize the available evidence about providers’ in-services and pre-service training methods and outcomes. This is especially relevant considering the modifications made in 2018 to the implementation guidelines of the Baby Friendly Hospital Initiative [28], in which the focus is not just in the hours of hands-on training but rather on the actual knowledge and abilities, which our study suggests can be at least partially met through virtual/online training.

Despite these positive findings, a key question regarding the semi-virtual training addressed in this study is whether it sufficiently increased problem-based and practical abilities for solving common breastfeeding problems [14, 29]. Considering the low levels of knowledge and abilities that healthcare providers showed at baseline, post-training scores clearly show that there is room for improvement to sustain and keep expanding their breastfeeding abilities, which are likely to require a more sustained practice training approach.

### Limitations

Given the nature of the research design, a potential limitation is participant selection bias in both cross-sectional surveys. Even though our sampling strategies were robust, this possibility cannot be discounted because we do not have information on those who did not answer the phone calls or with phones that were no longer in service. A second limitation is that this was not a randomized controlled trial because the EsIAN strategy was already being implemented when the training evaluation started. This implies that there can be confounding factors explaining differences in the outcomes (i.e. observed changes could be explained by exogenous factors unrelated to the intervention). However, we attempted to account for confounding at least in part through the propensity score matching statistical technique used. We acknowledge that this is not the traditional use of PSM (i.e. comparing non-randomized populations who got or not an intervention measured through cross-sectional surveys). In our case we had two independent cross-sectional random samples before and after the interventions. Given the independence of both samples, and that we explicitly removed individuals who were contaminated at baseline (i.e. already trained) and post-intervention (i.e. those who had not been trained), the analytical design complies with the PSM assumptions. Finally, another potential issue may have been caused by implementation issues linked to the training such as a weak face-to-face relationship with mentors from the first cascade, slow uptake of the virtual training, which have been reported in prior studies [8]. However, even considering these implementation challenges, improvements in breastfeeding knowledge were significant and large.

## Conclusion

Results showed that physicians and nurses in primary public healthcare clinics in Mexico had low baseline knowledge and abilities to promote and support breastfeeding. This fully justifies the recent recommendation proposed by an expert committee in Mexico regarding the need for increasing coverage and quality of breastfeeding in-service training of health professionals to address the dire breastfeeding situation in the country [2]. This study revealed that a semi-virtual, easy-to implement, in-service training improved knowledge and applied knowledge abilities relevant to breastfeeding among physicians, RNs, and nurse technicians in Mexico. The next step will be to assess whether these improvements among providers are positively affecting how they provide breastfeeding services and counseling to mothers and infants, most of whom live under conditions of poverty.

## Data Availability

The datasets used in this study are not publicly available but are available from the corresponding author on reasonable request.

## Abbreviations

ENSANUT: (for its acronym in Spanish) Mexican National Health and Nutrition Survey
EBF: Exclusive Breastfeeding
*EsIAN*: *Estrategia Integral de Atención a la Nutrición*
WHO: World Health Organization
INSP: (for its acronym in Spanish) National Institute of Health
RNs: Registered Nurses
